# The Communication of Timbral Intentions Between Pianists and Listeners and Its Dependence on Auditory-Visual Conditions

**DOI:** 10.3389/fpsyg.2021.717842

**Published:** 2021-09-21

**Authors:** Shen Li, Renee Timmers, Weijun Wang

**Affiliations:** ^1^School of Psychology, Central China Normal University, Wuhan, China; ^2^Key Laboratory of Adolescent Cyberpsychology and Behavior, Ministry of Education, Wuhan, China; ^3^Department of Music, The University of Sheffield, Sheffield, United Kingdom

**Keywords:** music communication, timbre perception, performer-listener chain, auditory-visual condition, cross-modal correspondence

## Abstract

The perceptual experiment reported in this article explored whether the communication of five pairs of timbral intentions (bright/dark, heavy/light, round/sharp, tense/relaxed, and dry/velvety) between pianists and listeners is reliable and the extent to which performers' gestures provide visual cues that influence the perceived timbre. Three pianists played three musical excerpts with 10 different timbral intentions (3 × 10 = 30 music stimuli) and 21 piano students were asked to rate perceived timbral qualities on both unipolar Likert scales and non-verbal sensory scales (shape, size, and brightness) under three modes (vision-alone, audio-alone, and audio-visual). The results revealed that nine of the timbral intentions were reliably communicated between the pianists and the listeners, except for the dark timbre. The communication of tense and relaxed timbres was improved by the visual conditions regardless of who is performing; for the rest, we found the individuality in each pianist's preference for using visual cues. The results also revealed a strong cross-modal association between timbre and shape. This study implies that the communication of piano timbre is not based on acoustic cues alone but relates to a shared understanding of sensorimotor experiences between the performers and the listeners.

## Introduction

Compared to the communication of emotions or expressiveness in musical performance, research conducted into the communication of timbral intention between performers and listeners is rare. From the performers' perspective, they intend to produce explicit timbres in their performances, which may lead to their self-satisfaction and sense of achievement (Holmes, 2011), relate to their holistic perception of expressive elements and music structure (Li et al., 2020), or be relevant to emotional expression (Juslin, 2000). In addition, piano teachers and students usually work on explicit timbral intentions in piano lessons using metaphors, gestures and modeling etc. to improve their communication of timbre-related performance goals (Li and Timmers, 2021). However, whether pianists can communicate timbral intentions reliably to the listeners under different auditory-visual presentation conditions remains unknown. An interview study (Li et al., 2020) on the conceptualization of piano timbre revealed pianists' extensive utilization of timbral intentions in piano performance; the findings also suggested that a pianist's concept of timbre is enriched by embodied experience, such as bodily preparations, indicating the relevance of visual cues. As a follow-up study, a perceptual experiment was conducted to examine the accuracy of communication of timbral intentions to listeners and its dependence on the visual and aural components of musical performance. It aims to explore the core question of how piano timbre is communicated, by using several sub-questions: Do pianists communicate timbral intentions to the audience and is the communication reliable? And what is the relevance of the auditory and/or visual components of a musical performance in timbre communication?

## The Research Background of Piano Timbre

To better understand the research background and research purpose, it is necessary to clarify the meaning and scope of timbre specified in this study, which we distinguished as “micro-perspective” and “macro-perspective.” In general, timbre studies have usually adopted a macro-perspective, which considers the differences in timbre produced from various sound objects (i.e., different sound sources). The “macro-perspective” focuses on the characterization of timbre of specific instruments or instrument groups. This could also concern differences within an instrument group (i.e., the timbre of piano A is different from that of piano B). An important aim may be to uncover the timbre space through which listeners categorize or distinguish instrument timbres (e.g., timbre intervals: McAdams, 2013). However, this study investigates piano timbre from a “micro-perspective”—which focuses on the timbral nuances produced from one instrument, from the point of view that a specific instrument can still have a variety of timbres depending on how the instrument is played (e.g., piano A has contrasting timbres when the key is either gently pressed or quickly struck).

One difficulty in the study of piano timbre lies in the interweaving of timbre and other performance parameters (e.g., intensity, articulation, and tempo) in the pianists' conceptions of piano timbre. It might be difficult for pianists to believe that, by controlling performed intensity, different ways of touching the keys have little effects on produced timbre—what they've changed are the attack noises (i.e., finger-key noise, key-keyframe noise; Goebl et al., 2014). Timbre may also be phrased as tone quality, tone color in the discourses of pianists.^1^ Sometimes we found these terminologies were used interchangeably in the writings and discourses of pianists, with the combined effect of timbre and other musical features being mentioned. Ortmann (1935) referred to “tone quality” and suggested that the perception of tone quality is subjective and results from our unified reaction to three variants: pitch, intensity, and duration. Bernays and Traube (2014) put forward the notion of *composite timbre*, referring to the complexity of piano timbre interwoven with other performance parameters when considering piano timbre in a musical and polyphonic context. Their study selected five verbal descriptors (dry, bright, round, velvety, and dark) and the pianists conceptualized and performed the given music pieces in accordance with these timbral nuances. For these reasons, this study investigates the beliefs and utilizations of piano timbre within the conventions of the pianistic community, rather than from an acoustic perspective. In other words, we will examine timbral communication in the context that pianists conceptualize their entire performance as expressing a timbral feature (i.e., mental conception: Kochevitsky, 1967) and rely on the coordination and adjustment of other musical parameters to align with the timbral intention.

## Music Communication Between Performer and Listeners

### The Role of Visual Information

Previous studies that investigated the role of visual information provided by the performer in communicating to the listeners mainly consider the communication of expressivity (Davidson, 1993, 1995; Broughton and Stevens, 2009) and emotional intentions (Dahl and Friberg, 2007). In Davidson's study (1993), a pianist was instructed to play a piece of music in three differently expressive ways (deadpan, projected, and exaggerated) and listeners were asked to rate the *expressivity* in the condition of seeing only, hearing only, or both seeing and hearing. This study found that once the pianist was playing with a certain degree of expressivity (i.e., in a projected and exaggerated manner), listeners could only differentiate between different degrees when the visual information was present and not in the audio-only presentation. Employing a similar experimental paradigm, Broughton and Stevens (2009) verified this result in the communication of expressivity in a marimba performance in which the audio-visual condition helped the listeners' differentiation of performances played with different expressive intentions compared to the audio-only condition. These studies suggested that the bodily movements and gestures of the performers provide identifiable information through which listeners can detect musical expressivity.

Performers express and communicate emotions to audiences [see a review by Juslin and Timmers (2010)] and visual information plays a role in the performer-audience communication chain (Camurri et al., 2003; Timmers et al., 2006; Dahl and Friberg, 2007) and for a duo partner (Wöllner, 2020). Dahl and Friberg (2007) examined whether the specific emotions (happy, sad, angry, and fear) expressed by the performer could be recognized by listeners using visual information only. This study confirmed that listeners (not necessarily musically trained) could easily recognize emotions such as happy and sad and were roughly accurate with anger, but failed to perceive fear. In a dancing-related study, Camurri et al. (2003) indicated that listeners can successfully detect a dancer's emotional intentions (e.g., joy, fear, grief, and anger) through movement cues only. These studies implied that even without the aural cues, visual cues are informative for listeners to recognize the emotional intentions of performers.

However, to the author's current knowledge, there is no existing research that investigates the communication of timbre in music performance and considers the visual effects of performers' gestures in this process. A possible exception may be a research project by Wapnick et al. (2004) who examined the visual perception of tone quality. The results indicated that the ratings of perceived tone quality together with the other five performance items (note accuracy, rhythmic accuracy, expressivity, adherence to style, and overall impression) were higher in the audio-visual presentation mode than the audio-only presentation. The present study aims to examine whether the communication of timbral intention between the pianist and the listeners is reliable and to what extent visual information influences judgment.

### Experimental Paradigm

#### Changing Audio-Video Presentation Modes

The first common approach in empirical studies of visual communication in music performance is to: (a) instruct the performer to play the same piece in a different manner (expressive vs. non-expressive, or with different emotional intentions) and (b) vary the mode of audio-visual presentation of the recordings and investigate its influence on the listeners' evaluation of the performance. Relevant research has been conducted to investigate the role of vision in the evaluation of emotional engagement (Timmers et al., 2006), performance quality (Wapnick et al., 2000, 2004), and expressive intention (Davidson, 1993, 1995). Similarly, visual information from a singer's facial expression (e.g., eyebrow raised or not, eyes widened or not, lip movement) affected a listener's judgment of the emotional valence of a music piece (Thompson et al., 2005).

#### Employing Congruent/Incongruent Pairings

Another approach employed by researchers is to make congruent or incongruent pairings of audio-video stimuli and examine their impact on listeners' responses. This approach helps to understand the impact of auditory-visual integration on participants' processing of new music stimuli, as the “McGurk” effect demonstrated in the studies of speech communication (McGurk and MacDonald, 1976). Relevant research has been conducted to investigate the extent to which vision influences the processing of tone duration (Schutz and Lipscomb, 2007), pitch interval (Thompson et al., 2005; Thompson and Russo, 2007), and timbre (Saldaña and Rosenblum, 1993). For example, Thompson et al. (2005, Experiment 3) examined the influence of (in)congruent pairings of singers' facial expressions with singing clips on the perception of interval size and found that seeing an incongruent pairing (e.g., a small melodic interval accompanied by images of singing a larger interval) resulted in ratings of a larger interval than either of the original pairings. The perceptual experiment conducted by Schutz and Lipscomb (2007) demonstrated that the visual perception of stroke action (seeing either longer or shorter physical gestures) in a marimba performance influences listeners' perception of tone duration. In addition, Saldaña and Rosenblum (1993) examined the perception of cello timbre influenced by the visual information of stroke action (plucking/bowing) in cello performances. Their studies found that watching a video performance enabled the listeners to distinguish between plucking and bowing and that seeing a bowing movement led to higher ratings of bowing timbre and seeing a plucking action resulted in a larger plucking timbre response.

The above studies suggest that we could either manipulate the presentation mode of one stimulus, or combine two stimuli that convey different timbres. This study will use the first approach (changing aural-visual presentation modes) to examine the visual communication of timbral intentions between pianists and listeners, due to the ease of operation and the study purpose. Playing with different timbral intentions leads to a variety of performance parameters e.g., timing and performance speed, which result in the difficulty of synchronizing audio with unmatched video in the “artificial” audio-visual stimuli (i.e., incongruent pairing). Instead, the second approach will be more effective in examining the perception of single piano tones or chords, but the focus of this research is the polyphonic musical background.

There are increasing numbers of scholars (Dahl and Friberg, 2007; Behne and Wöllner, 2011) investigating in detail which visual aspects and which specific regions of bodily movements have an impact on listeners' music perception, instead of looking performers' bodily movements as an integrated part. For example, ancillary gestures—those that are not required for producing sounds (Wanderley et al., 2005; Wanderley and Vines, 2006), have been found to have an impact on audiences' perception of musical tention and phrasing (Vines et al., 2006). Sound-producing gestures—those movements that are effectively produce sounds (e.g., the hands of a marimba player, or the lips of a singer; Jensenius et al., 2010), can influence listeners' judgement of musical notes at a perceptual level (see section Employing Congruent/Incongruent Pairings). Dahl and Friberg (2007) tested the influence of viewing condition (full, nohands, nohead, and only-head) on the detection of emotions. They found that happy emotion was better detected when seeing upper body movement while sad emotion was better recognized when the head movement information was available. Seeing the whole body led to the highest accuracy of recognized emotions. These studies suggested that we could combine two angles in the video recordings—one focused on the movement of the upper body and the head, and the other one focused on the sound-producing gestures (i.e., finger movements).

## An Embodied Perspective on the Communication of Timbral Intentions

### From an Information-Processing Model to an Embodied Model

The rationale of communicating a timbral intention from the performer to the listeners can be explained by several well-known models, for instance, the information-processing model (Kendall and Carterette, 1990) and the lens model (Juslin, 1998, 2000; Juslin and Lindström, 2010). According to Kendall and Carterette (1990), the information-processing model of music communication addresses how the composer and performer convey their expressive intentions and how the listener perceives the intended message from the composer/performer *via* message recoding. The composer's encoding process goes from ideation to notation; the performer then recodes the notation into acoustic signals and, finally, these acoustic signals are recognized by listeners and result in ideation. In this context, timbral intentions work as a message that the performer intends to communicate to their audience and the encoding and recoding of this timbral message relies on a successful recognition of acoustic cues. For acoustic cues, Juslin and Lindström (2010) adapted the lens model from seminal work Brunswik's(1956) and applied it to the musical context. The modified lens model explains the utilization of cues (i.e., tempo, loudness, articulation etc.) in the communicative process of specific emotions between the performer and the listeners, which suggests that a successful communicative process happens when the performer's cue utilization matches that of the listeners. Both models have acknowledged the feasibility of communicating implicit messages (i.e., emotions) within the musical communication process; however, these models seem to regard musical communication purely as a sonic art, as can be seen in Juslin and Lindström's model of acoustic cues as a lens of musical expression, as well as Kendall and Carterette's model which regards the acoustic signal as transmitting the composer/performer's thoughts to the listener's ideation.

The theory of embodied cognition has prompted the understanding of listeners' musical experience, as revealed by the following research. For example, Davidson and Correia (2002) highlighted the role of bodily movements in shaping the listeners' perception of musical expressiveness. More importantly, the common knowledge of bodily experience i.e., balance, scale, force, and cycles (Johnson, 1987) forms the common ground on which performers and audiences can communicate and appreciate the musical work (Davidson and Correia, 2002). Leman (2008) extended the previous research and proposed a model of embodied music communication based on the decoding and encoding of motor control. The performer realizes the musical goal/idea through corporeal articulations, while the instrument is the mediation which may form a closed loop with the performer with haptic, sonic and visual feedback whilst also transmitting the sonic and visual energy to the listener. Listeners can make sense of the communicative process through a mirror process i.e., a corporeal and cerebral understanding of the intended actions. Leman's (2008) model suggests multimodal sensing of music communication and extends the understanding of musical communication from the decoding/encoding of sonic forms to include those of motor control.

It is worth noting that listeners' embodied listening experience does not merely incorporate the visual perception of musical performance, it could involve more covert imitation of sound-producing actions. Godøy (2006) related the notion of gestural-sonorous object to musical sounds, referring to the phenomenon that humans would mentally trace sound as a continuous process (i.e., the onsets, contours, textures and envelops involving hands, fingers, arms etc.) in the perception or imagination of musical sounds. Cox (2016) proposed the mimetic hypothesis and paid attention to the covert mimetic behavior (MMI, mimetic motor imagery) besides the overt imitation of performance actions (MMA, mimetic motor action). He suggested that, MMI plays important roles in the comprehension of musical sounds, for instance the instrumental timbre—despite the limitations of human voice to overtly imitate other instrumental timbres, “what matters is the attempt to emulate the sound, to feel something of what it would be like to make such sounds, and to thereby feel something of what it would be like to be an entity capable of making such sounds” (p. 32).

### Sensorimotor Perceptions in Timbral Communication

The embodied model of music communication implies that timbral communication may be associated with sensorimotor perceptions, as indicated by several studies (De Poli et al., 1998, 2017; Baraldi et al., 2006). For example, De Poli et al. (2017) claimed that sensorimotor expressivity is the part of musical expressivity that is not covered by musical and emotional expressivity and that it reflects certain cross-modal correspondence (CMC) features. They further developed an FEI (friction, elasticity, and inertia) metaphor that characterizes the sensorimotor expressivity in a kinetic-energy two-dimensional space (De Poli et al., 2009) and demonstrates that participants can describe perceived expressivity *via* FEI metaphors (e.g., hard-soft, light-heavy categories).

Timbre communication may also involve CMC features (Wallmark and Kendall, 2018; Wallmark, 2019a,b). For example, timbre metaphors such as bright/dark, rough/smooth reflected cross-modal correspondence with vision and touch (Wallmark, 2019b). Timbre metaphors in CMC categories encompass “an embodied conceptual transfer process by which an auditory target domain (timbre) is understood in reference to a non-auditory source domain (vision, touch, taste, and smell)” (2019a, p. 594). Therefore, we predict that the listeners in our study will describe perceived timbral qualities in piano performances by referencing multimodal sensations.

### Communicating Timbre: Emotion and Musical Expressivity

In the field of music emotion studies, timbre is closely related to the perceived emotions of listeners (Gabrielsson and Juslin, 1996; Balkwill and Thompson, 1999; Hailstone et al., 2009) as well as the experienced emotions of performers during music performance (Holmes, 2011; Van Zijl and Sloboda, 2011). For example, the selection of instrumental timbre effectively influences listeners' judgment of emotion conveyed by the music (Balkwill and Thompson, 1999). With a systematic control on other performance parameters (loudness, tempo, and melody), there is still a robust effect induced by timbral feature (e.g., spectral content, attack) on the ratings of listeners' perceived emotion (Hailstone et al., 2009). Juslin and Timmers (2010) suggested that: a bright timbre is used for expressing happiness; a soft timbre is suitable for expressing tenderness; a dull timbre is good for expressing sadness; a sharp timbre is appropriate for expressing anger. From a performer's perspective, producing timbral nuances is not only about playing techniques, but is inseparable from musicians' own emotions experienced during music practice/performance. Holmes (2011) suggested that the employment of specific timbre is the key element of musicians' internal motivation to make a successful performance during which process their felt emotions are mainly feelings of satisfaction and elation. Van Zijl and Sloboda (2011) found that focusing on 'sound/tone color' is one of the key elements of the inner technique toward an expressive music interpretation, and the process of focusing helped them to bring their own emotions in line with the musical emotions.

**Figure 1:**
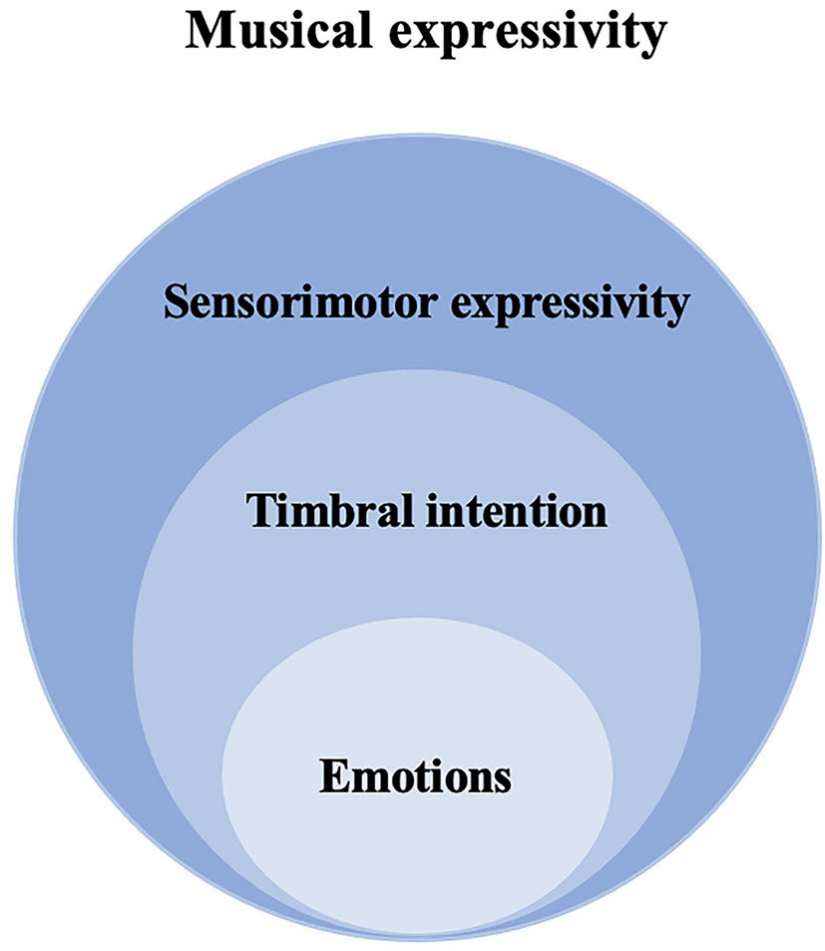
Logic relationship between timbre, emotion, and musical expressivity.

The above research seems to suggest that the expression of timbral nuances, driven by an affective intention of the performer, appears to be an accessory of emotional communication in musical performance. However, this study would rather consider timbral communication not under the umbrella of emotional communication, but within the framework of musical expressivity (as shown in Figure 1). Our previous research (Li and Timmers, 2021) found that playing a short musical phrase or an entire section with specific timbral intention without indicating any relevance to affective intention is naturalistic and feasible. The study results revealed that the piano teachers and students usually worked on an entire musical phrase with a consistent timbral intention (e.g., “lute-like timbre,” “timbre of princess and earl”) and employed multimodal communication strategies (e.g., modeling, verbal explanation, physical touch, gesture, etc.) to achieve these timbre-related goals. According to Bernays and Traube (2014), verbal instruction of basic emotions and timbral intentions are not comparable, and the vocabularies of describing piano timbre are consensual and meaningful to pianists (Faure, 2000; Bellemare and Traube, 2005). The study of timbre semantics (Wallmark, 2019a) suggests that the description of timbre impression can include many possibilities beside emotion, including acoustics, cross-modal correspondence (CMC), mimesis, action, etc.

Timbre, as an important musical message which conveys musical expressivity between performers and listeners, has received scholarly attention (Juslin and Laukka, 2003; Barthet et al., 2010). Barthet et al. (2010) found that changes in timbral features across expressive levels in a clarinet performance did not happen at every note but were specific to some notes or groups of notes or specific musical passages. When cellists were asked to make either expressive performance or physically constrained performance (Rozé et al., 2017), the timbre was modified in the constrained postural condition. The above studies imply that the communication of timbral intention in piano performance might involve not only affective purposes but working as an important musical message when conveying musical expressivity from performers to listeners. As mentioned before, sensorimotor expressivity is the core component of the embodied communication of musical expressivity.

### The Current Research

This study aims to examine the communication of piano timbre between pianists and listeners and the influence of audio-visual presentation modes on the communication outcome. The focus on piano timbre originates from the touch-tone debate in piano performance, corresponds the trend of increasing attention on semantic studies of piano timbre (Bellemare and Traube, 2005; Bernays, 2013; Kojucharov and Rodà, 2015), and sheds light on the understanding of the relationship between timbre and musical expressivity (Barthet et al., 2010; Ystad et al., 2019). This research expects that, like expressivity and emotions, pianists can express different timbral intentions in their performances and listeners can detect those intentions from either the variations in sound or the visual cues in performative gestures (H1). We also expect that the listeners' ratings of perceived timbre in response to performances with contrasting timbres will be significantly different (H2). Concerning the visual influence, we expect that audio-visual presentation modes of the music performances will influence the communication of timbral intentions (H3).

Hypothesis 1 (H1): the communication of timbral intentions is reliable and successful in the performer-listener communication chain.
Hypothesis 2 (H2): the main effect of heard timbre on timbre ratings: The listeners can successfully differentiate between performances with contrasting timbral intentions.
Hypothesis 3 (H3): the interaction effect of AV condition and heard timbre: the differentiation between contrasting timbral intentions will be influenced by the auditory-visual presentation modes.

We also developed 10 timbre descriptors (see Table 1 below) as the perceptual scales for participants in the current perceptual experiment. Bright, dark, round, velvety, and dry were selected from the study of Bernays and Traube (2014),^2^ whilst the others are either their antonyms (sharp) or new descriptors in pairs (heavy/light, relaxed/tense). These 10 timbre metaphors aim to examine the multidimensionality of timbre perception. The heavy and light descriptors represent a kinetic experience associated with piano timbre perception; relaxed and tense relate to the muscular sensations when responding to piano timbre. It is noteworthy that the contrast between “velvety” and “dry” was not noticeable; however, they described the characteristics of an object's surface and represent different types of tactile feelings.

**Table 1:** Ten timbre descriptors used in the perceptual experiment.

	**Timbre descriptors**	
Pair 1	Bright^*^	Dark^*^
Pair 2	Round^*^	Sharp
Pair 3	Light	Heavy
Pair 4	Relaxed	Tensed
Pair 5	Velvety^*^	Dry^*^

* *refers to the timbre descriptors borrowed from Bernays and Traube's (2014) study*.

**Figure 2:**
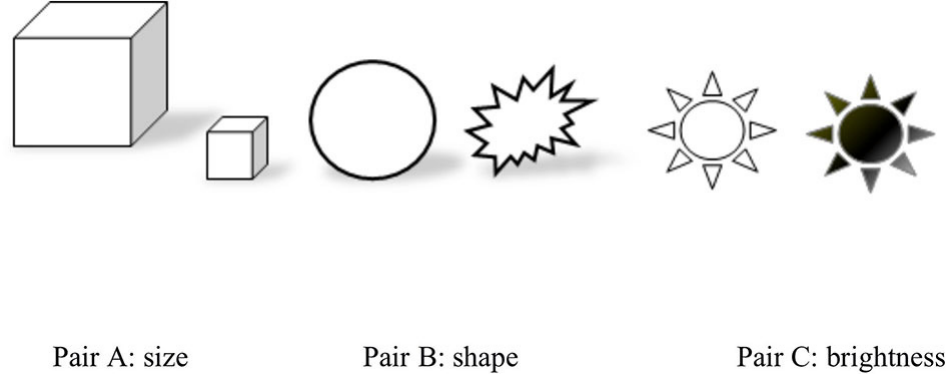
The contrasting images employed in this study to examine cross-modal correspondence (CMC) related to piano timbre perception.

In addition to the 10 metaphors, we employed non-verbal perceptual scales (i.e., image choice) to directly test CMC features with musical timbre i.e., three pairs of images that show contrasting brightness, shape, and size (see Figure 2). Timbre-shape and timbre-brightness associations have been found: a soft timbre (e.g., piano) is strongly associated with round shapes together with blue, green, or lighter grayscales, whereas a harsh timbre (e.g., crashing cymbals) is associated with angular shapes together with red, yellow, or darker grayscales (Adeli et al., 2014). Additionally, timbre can be associated with visual textures that vary in terms of sharpness, compactness, and sensory dissonance (Giannakis, 2006). Therefore, this study proposes

- Hypothesis 4: the timbre expressed by the performer will significantly influence the listeners' choice between contrasting images.

## Experiment

### Participants

*Performers*. Three female Chinese pianists (age 23–27) from the Department of Music at the University of Sheffield were asked to give performances to create the stimuli used in the listening experiment. They were all majoring in piano performance studies (one Ph.D. student, two Master's students) and regularly performed classical, baroque and contemporary music as part of their performance repertoire.

*Listeners*. Twenty-one music students (19 females and two males; Mean age = 21.89, SD = 2.03) from the music department at the University of Henan in China participated in the listening experiment. Fourteen of them were undergraduates and seven of them were postgraduates. All participants were majoring in piano performance studies. Based on large effect sizes found in the three-way interaction (Partial Eta Squared 0.15 is considered large, Cohen, 1988), we conducted a post hoc power analysis using G power 3.1 (Faul et al., 2007), that determined we had 99% power to detect large effects in a three-way interaction with 5% Type I error rate.

### Stimuli

**Figure 3:**
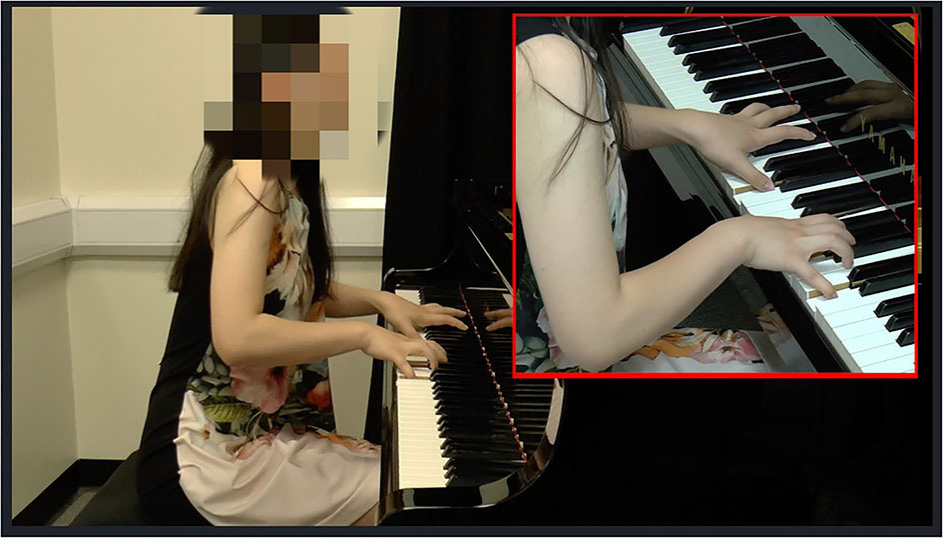
The video presentation with two camera angles.

In total, there were three musical pieces x 3 AV presentations x 10 instructed timbres totaling 90 music excerpts. The three musical pieces was performed by three different pianists (piece 1 pianist 1, piece 2 pianist 2, piece 3 pianist 3), and each musical piece was played with 10 types of timbral intentions, which were audio- and video-recorded. The video recordings were captured from two angles: a global view that showed the sitting pianists from the side (i.e., the viewing of ancillary gestures), and a more local view that was focused on the hands and finger (i.e., the viewing of sound-producing gestures; see Figure 3).

In the preparation of experimental stimuli, the three pianists practiced and recorded all three musical pieces with 10 timbres, but only one musical piece played by each pianist was selected for inclusion in the listening experiment to avoid boredom and the effect of repetitive listening on listeners' responses (Morimoto and Timmers, 2012). Otherwise, the listeners had to listen to the same musical piece 30 times (three performers playing one musical piece repetitively using 10 different timbres). When matching the performer and the piece, we considered the criteria of fewer mistakes and more expression in the performances of each pianist to optimize the quality of the musical material presented to listeners.

The three pianists' performances were recorded on a Yamaha grand piano (Disklavier Pro S6) in the Sound House Studio of the Department of Music at the University of Sheffield. Panasonic HC-V770k HD Camcorder and Tascam DR-05 Audio Recorder Kit were used to obtain higher recording quality.

Three pianists were given the pieces [selected from Bernays and Traube (2014), see Appendix 1] 1 month before the recording and each of them experienced a recording trial before the actual recording to enable them to get used to the cameras, the procedure, and the piano. The practice time also allowed them to arrive at the appropriate performance manner for each special timbre. They were told to play the piece with a consistent timbral intention and performance instructions were explained in both written (see Appendix 3) and oral forms. To ensure that pianists played in response to timbral intentions rather than emotional intentions, the objectives of recording their performances were clearly explained to them. The three pianists were told that their performances would be used as stimuli in a perceptual experiment and the listeners' focus would be perceived timbral quality. All three pianists have participated in the previous interview study on piano timbre, which gave them and the researchers a common understanding of the research objectives. The original musical pieces borrowed from Bernays' study do not have any expression, dynamic, articulation, phrasing, and accent markings, which gave the performers scope to vary performance parameters to achieve a desired tone quality.

### Procedure

The experiment tested participants individually by presenting the recordings on a computer monitor. After the stimuli presented, 21 participants were asked to rate the presence of the 10 timbres on a 1–9 unipolar Likert scales (task 1) and to choose from three pairs of non-verbal sensory scales (task 2). We considered using unipolar scales instead of categorical responses, allowing for ambiguous and relative open responses (e.g., both bright and light might be perceived to certain extent, or none of the timbres might be strongly perceived). Each music excerpt lasted around 15 s and the participants were required to complete two tasks for each excerpt intuitively.

Participants gave their responses on a paper-based response sheet (see Appendix 2, two tasks in responses to each excerpt). Response sheet was written in both English and Chinese. Although there might be potential differences between Chinese and English timbre descriptors (Namba et al., 1991), the participants reported no difficulty in understanding 10 timbre descriptors which were also commonly used in Chinese context. The ordering of musical excerpts was randomized to ensure that the same timbre could not occur more than twice consecutively, and we prepared two different random orderings for counterbalancing. The monitor had a 15″ screen and was placed in front of the participants within a comfortable viewing distance (80–100 cm). The experiment contains two practice trials and 90 experiment trials. Recordings were presented over headphones at a comfortable level. Participants were encouraged to respond intuitively after experiencing the stimuli for the first time, but they could play the recording repeatedly if they asked, or they were hesitant about the answer. The majority of them played the stimuli just once and responded quickly, but two out of 21 participants took half hour longer than others due to unsure answers and replay of stimuli. The duration of the entire experiment normally took between 45 min to an hour. In the middle of the experiment, there was a short break.

## Results

To examine how well the performers communicated the timbre to the participants, we calculated the percentage of correct answers in the ratings (perceived timbre) for each target timbre (heard timbre). A one-sample t-test was conducted to test whether the number of correct answers was above the chance level (10%). Timbre ratings were then entered into a three-way ANOVA with repeated measures for heard timbre (two levels: target, opposite), AV condition (three levels: AO, AV, and VO), and pianist/piece (three levels: P1, P2, and P3). To simplify the analysis and assure statistical power, the ability to communicate a particular timbre was tested per timbre pair rather than by comparing across all 10 timbre levels individually. Finally, PCA was conducted to explore which of the 10 timbres capture the same variance, with the inclusion of acoustic and visual information for further comparison.

### Percent Correct Score

We calculated the percentage of correct answers for each target timbre by re-coding data as either 1 (correct) or 0 (incorrect). The percent correct scores were then further divided into an absolute percentage and a relative percentage.^3^ For example, in the perception of the dark timbre, only 15.34% of listeners gave the highest ratings for darkness when exposed to an excerpt performed with *dark*^4^ timbre, and the score was even below chance level when seen without presentation of audio (VO: 9.52%). However, using relative percentage correct, the success of communication of darkness is above chance (19.15%): listeners' rating of darkness was relatively high for this target timbre compared to when asked to perform the same music with a different timbre.

The results of the percent correct answers for each target timbre are summarized in Table 2 below, including the specific scores in each audio-visual condition and the average score. These results indicate that percent correct responses for *dark, round*, and *bright* timbres were the lowest (dark < round < bright), and percent correct responses for *light, relaxed*, and *sharp* timbres were the highest (sharp>relaxed>light). It is noteworthy that the relative percent correct answers regarding round timbre and sharp timbre was considerably higher than the absolute percentage. This implies that the performed piece did not sound very round or sharp, but performers were able to change the degree to which those excerpts were perceived as round or sharp. When considering the influence of AV stimuli on the absolute/relative percent correct score, *tense* and *relaxed* timbre were more likely to be rated as high when visual information was presented—as can be seen by the higher percent correct score in both absolute and relative percentages in the audio-visual and the visual-only condition compared to the audio-only condition. The impact of the AV stimuli on other timbre evaluations was more variable, the details of which will be investigated in the next analysis of variance of the timbre ratings.

**Table 2:** Percentage of listeners' correct answers for a target timbre (values in each AV condition and their average).

**Rated timbre**	**Absolute percentage** ^**a**^				**Relative percentage** ^**b**^			
	**Average**	**AO**	**AV**	**VO**	**Average**	**AO**	**AV**	**VO**
Dark	**15.34**	12.70	23.81	9.52	**19.15**	17.49	23.33	17.46
Round	**19.15**	13.33	20.63	22.22	**32.98**	28.33	36.51	31.75
Bright	**23.4**	28.57	20.63	21.67	**29.26**	23.81	31.75	31.67
Velvety	**24.34**	23.81	22.22	26.98	**29.63**	26.98	30.16	31.75
Tense	**26.98**	19.05	30.16	31.75	**25.4**	20.63	25.40	30.16
Dry	**27.13**	31.75	26.98	21.67	**23.94**	25.40	22.22	25.00
Heavy	**31.91**	36.67	28.57	30.16	**30.32**	38.33	28.57	25.40
Light	**34.92**	30.16	36.51	38.10	**39.15**	39.68	42.86	34.92
Relaxed	**35.45**	31.75	34.92	39.68	**34.92**	28.57	38.10	38.10
Sharp	**39.68**	47.62	42.86	28.57	**52.38**	61.90	55.56	39.68
Average	**27.83**	27.54	28.73	27.03	**31.71**	31.11	33.45	30.59

a *When the rating of the target timbre was higher than ratings of the other nine timbres when the listener was presented with the performance of a target timbre*.

b *When the rating of target timbre was relatively higher for the performance with target timbre than in the other performances of a piece*.

To examine whether the evaluation of each timbre was above chance level, a one-sample *t*-test was conducted, in which the test value was 0.1. This is a chance probability that a target timbre is the highest rated timbre. The values of t, df, and mean score for the one-sample t-test are summarized below (Table 3). This analysis showed that the mean values of all variables were significantly higher than the chance level of 10%, which gives the first indication of a reliable communication of the target instruction. Thus, the first hypothesis (H1) was supported. Interestingly, for nine timbres the communication was even successful in the visual-only condition, except for the *dark* timbre (9.52% in VO condition).

**Table 3:** *T*-value, *p*-value, and mean score of absolute percent correct in the one-sample *t*-test.

**Rated timbres**	**Mean**	***t*-value**	**df**	***p*-value**
*Dark* percent correct	0.15	2.03	188	<0.05
*Round* percent correct	0.19	3.18	187	<0.05
*Bright* percent correct	0.23	4.33	187	<0.01
*Velvety* percent correct	0.24	4.58	188	<0.01
*Tense* percent correct	0.27	5.25	188	<0.01
*Dry* percent correct	0.27	5.27	187	<0.01
*Heavy* percent correct	0.32	6.43	187	<0.01
*Light* percent correct	0.35	7.17	188	<0.01
*Relaxed* percent correct	0.35	7.30	188	<0.01
*Sharp* percent correct	0.40	8.32	188	<0.01

### Three-Way ANOVA Results

In the repeated measures three-way ANOVA, the main effect of heard timbre (two levels: target timbre, opposite timbre) is a key indicator of successful timbral communication, as the score indicates the differences in timbre ratings across two performances with two contrasting timbres (target, opposite). In other words, significant differences suggest that listeners can successfully recognize performers with target timbres, otherwise performances with target timbre and opposite timbre are confounded. The overall ANOVA results are in Table 4.

**Table 4:** Three-way repeated measures ANOVA main effects and interactions for listeners' evaluation of 10 timbres.

**Evaluation**											
**Effects**		**Brightness**	**Darkness**	**Heaviness**	**Lightness**	**Roundness**	**Sharpness**	**Relaxed**	**Tense**	**Dry**	**Velvety**
Timbre	**F**	22.88^**^	3.92	59.02^**^	52.44^**^	32.38^**^	45.38^**^	39.05^**^	45.30^**^	31.18^**^	70.25^**^
	ηp^2^	0.77^^**^^	0.2	0.76^^**^^	0.73^^**^^	0.66^^**^^	0.73^^**^^	0.66^^**^^	0.70^^**^^	0.62^^**^^	0.79^^**^^
	df	(1, 16)	(1, 16)	(1, 19)	(1, 19)	(1, 17)	(1, 17)	(1, 20)	(1, 20)	(1, 19)	(1, 19)
Pianist/ Piece	**F**	11.90^**^	31.48^**^	73.12^**^	57.28^**^	6.80^*^	10.25^**^	8.42^**^	12.83^**^	8.93^**^	17.22^**^
	ηp^2^	0.43	0.66^**^	0.79^**^	0.75	0.53^*^	0.61	0.30^**^	0.39^**^	0.32^**^	0.48^**^
	df	(2, 32)	(2, 32)	(2, 38)	(2, 38)	(2, 34)	(2, 34)	(2, 40)	(2, 40)	(1, 19)	(1, 19)
AV stimuli	**F**	0.78	4.32^*^	3.81^*^	0.91	11.56^**^	14.75^**^	0.48	1.39	1.02	4.37^*^
	ηp^2^	0.05	0.21^*^	0.17^*^	0.05	0.64^**^	0.68^**^	0.02	0.07	0.05	0.19^*^
	df	(2, 32)	(2, 32)	(2, 38)	(2, 38)	(2, 34)	(2, 34)	(2, 40)	(2, 40)	(2. 38)	(2. 38)
Timbre ^*^ Piece	**F**	0.93	0.86	1.28	7.96^**^	11.31^**^	8.21^**^	8.11^**^	3.13	2.85	3.84^*^
	ηp^2^	0.06	0.05	0.06	0.30^**^	0.63^**^	0.57^**^	0.29^**^	0.14	0.13	0.17^*^
	df	(2, 32)	(2, 32)	(2, 38)	(2, 38)	(2, 34)	(2, 34)	(2, 40)	(2, 40)	(2. 38)	(2. 38)
Timbre ^*^ AV	**F**	0.28	0.83	0.17	0.11	0.11	1.09	3.81^*^	6.95^**^	2.29	3.80^*^
	ηp^2^	0.02	0.05	0.01	0.01	0.01	0.06	0.16^*^	0.26^**^	0.11	0.17^*^
	df	(2, 32)	(2, 32)	(2, 38)	(2, 38)	(2, 34)	(2, 34)	(2, 40)	(2, 40)	(2. 38)	(2. 38)
Piece ^*^ AV	**F**	1.86	1.36	2.21	0.91	2.22	1.55	3.10^*^	0.53	0.62	0.41
	ηp^2^	0.10	0.08	0.10	0.05	0.12	0.08	0.13^*^	0.03	0.03	0.02
	df	(4, 64)	(4, 64)	(4, 76)	(4, 76)	(4, 68)	(4, 68)	(4, 80)	(4, 80)	(4, 76)	(4, 76)
Timbre^*^ Piece ^*^ AV	**F**	0.31	1.16	4.80^**^	1.20	1.52	4.04^*^	1.83	0.73	0.75	3.31^*^
	ηp^2^	0.02	0.07	0.45^**^	0.06	0.08	0.19^*^	0.08	0.04	0.04	0.15^*^
	df	(4, 64)	(4, 64)	(4, 76)	(4, 76)	(4, 68)	(4, 68)	(4, 80)	(4, 80)	(4, 76)	(4, 76)

** *p < 0.01*;

* *p < 0.05*.

*Bold values indicate the significant results in the main effects and interaction effects*.

#### Main Effects

We found the main effect of heard timbre for all timbre ratings except for the *dark* timbre, indicating that the listeners failed to recognize performances with *dark* timbre regardless of who was performing and in which condition. This result is in line with the results in section Participants, which show that the percentage of correct answers for *dark* evaluation is the lowest (15%) among the 10 timbres. Therefore, H2 was mostly supported - the listeners can successfully differentiate between performances with contrasting timbral intentions except when the music was played with dark timbre.

#### Two-Way and Three-Way Interaction

**Figure 4:**
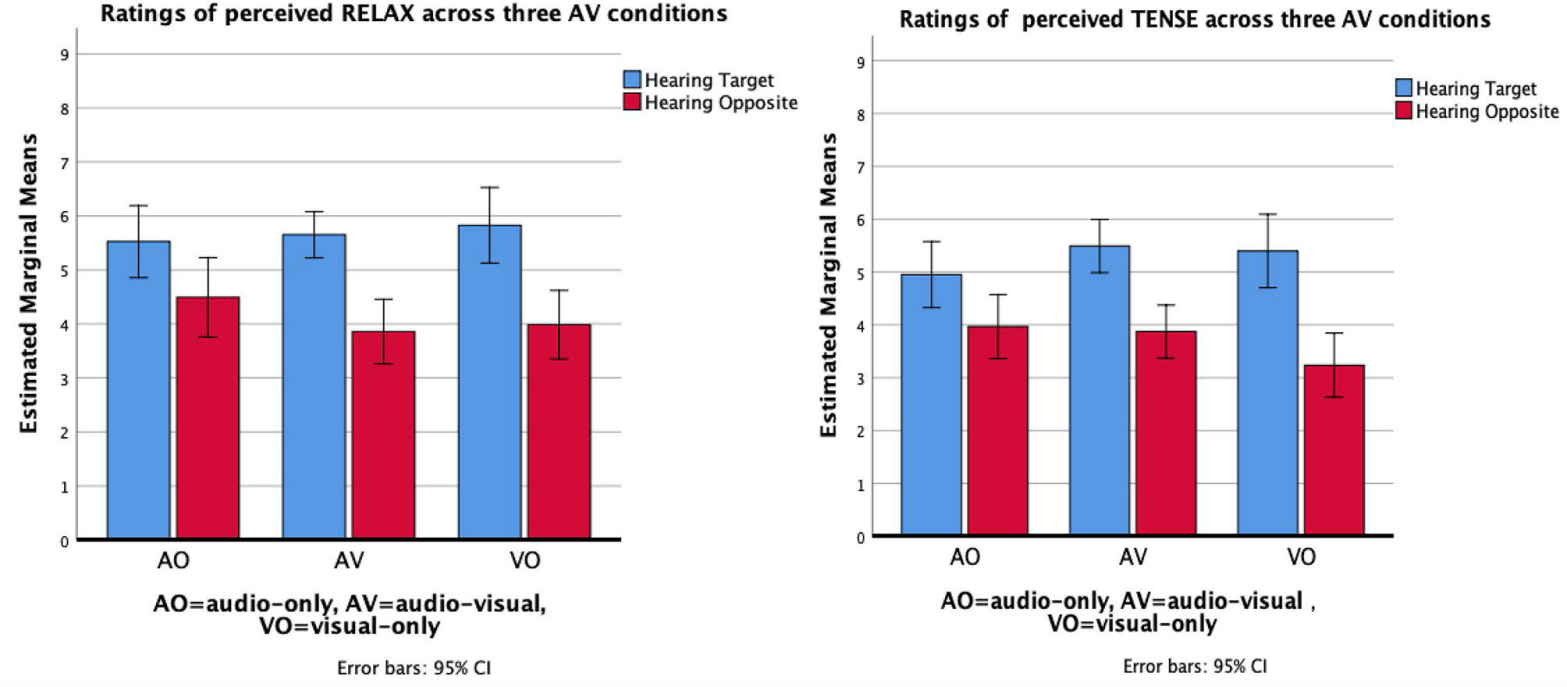
Mean values of the evaluation of *Relaxed* timbre **(left)** and *Tense* timbre **(right)** across three AV stimuli found by averaging the pianist/piece factor. Error bars represent confidence intervals.

**Figure 5:**
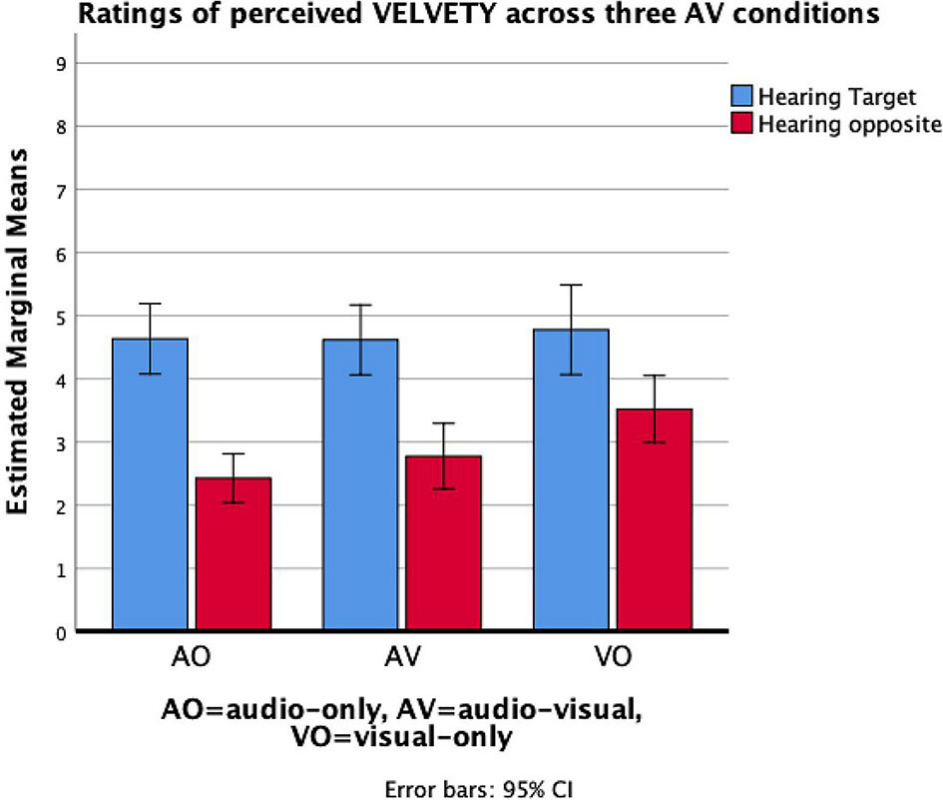
Mean values of the evaluation of *Velvety* timbre across three AV stimuli found by averaging in piece/performer factor. Error bars represent confidence intervals.

*Heard Timbre* × *condition*. The two-way interaction between heard timbre and condition (three level: AO, AV, and VO) suggests that the recognition of a target timbre is influenced by auditory-visual presentation modes. We found a significant interaction effect between heard timbre and AV condition for the communication of three timbres: *relaxed, tense*, and *velvety*. For *relaxed* timbre evaluation, planned contrast analysis between conditions contains vision and sound-only condition suggested that the communication was better when both audio and vision contained than the sound-only condition, *F*_(1, 20)_ = 15.56, *p* < 0.01, ηp^2^ = 0.44. For *tense* timbre evaluation, planned contrast results indicated that tension was differentiated more clearly in the AV conditions than in the audio-only condition, *F*_(1, 20)_ = 5.00, *p* < 0.05, ηp^2^ = 0.20. Figure 4 displays the evaluation for relaxed timbre (left) and tense timbre respectively, where the interval (i.e., differences in ratings across two performance with target timbre and opposite timbre) was larger in visual conditions. However, the participants differentiated the *velvety* timbre more clearly in the audio-only condition than in the audio-visual condition, *F*_(1, 20)_ = 9.10, *p* < 0.01, ηp^2^ = 0.31 (Figure 5).

**Figure 6:**
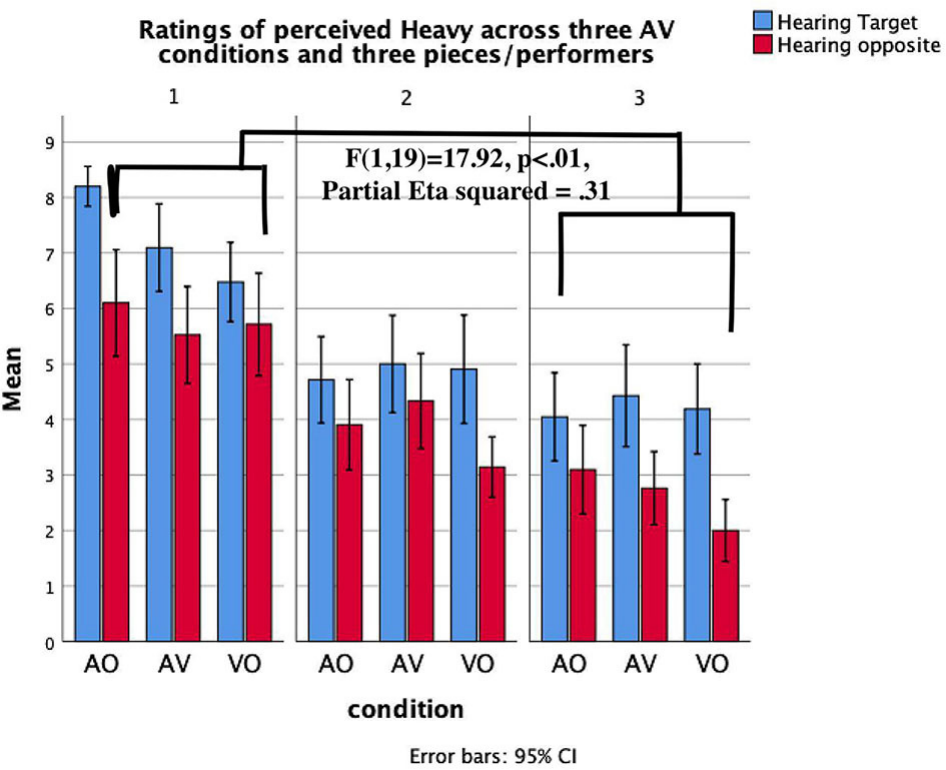
Mean values of the evaluation of *Heavy* timbre across three pianists/pieces and AV stimuli. Error lines represent confidence intervals.

**Figure 7:**
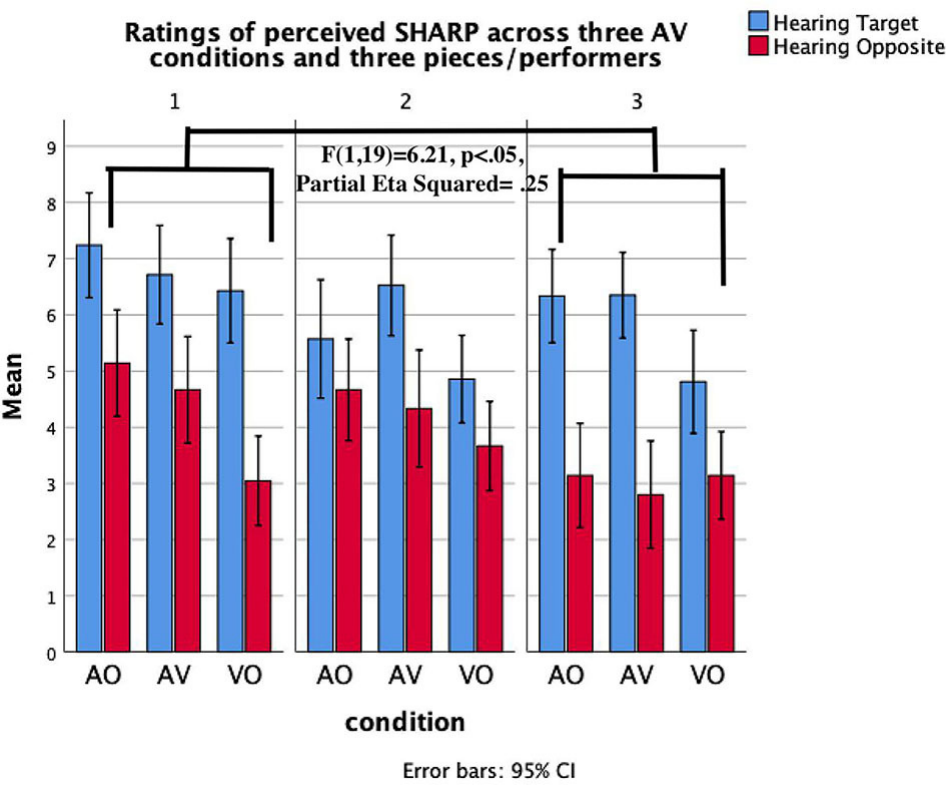
Mean values of the evaluation of *Sharp* timbre across three pianists/pieces and AV stimuli. Error lines represent confidence intervals.

**Figure 8:**
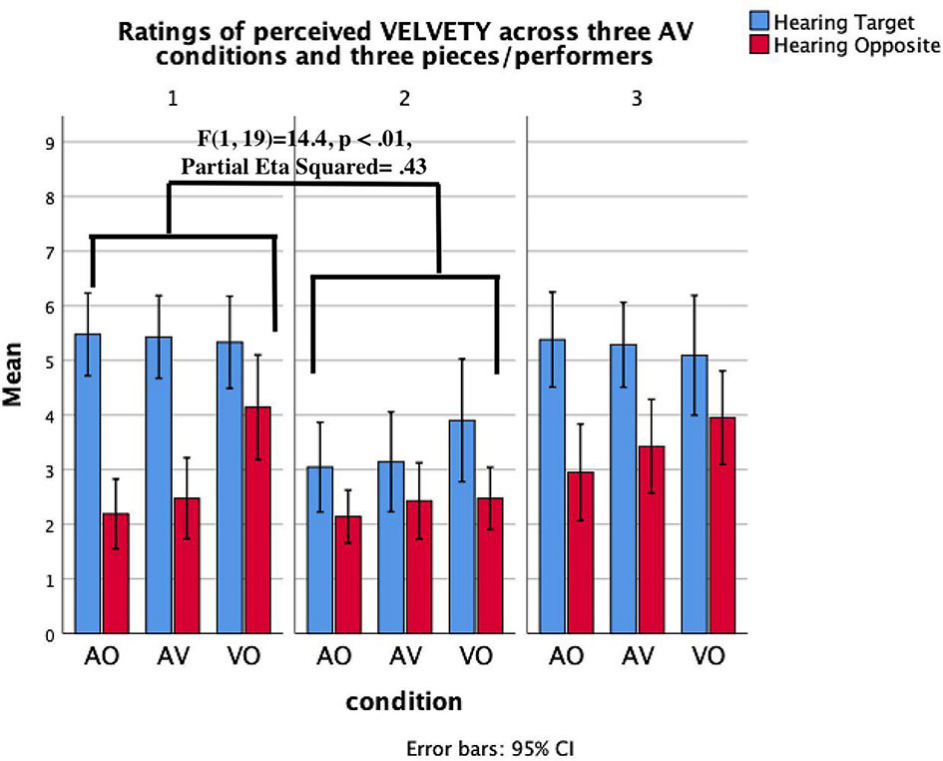
Mean value of the evaluation of *Velvety* timbre across three pianists/pieces and AV stimuli. Error lines represent confidence intervals.

*Heard Timbre* × *condition* × *piece****/****performer*. The three-way interaction between heard timbre, condition, and piece/performer suggests the extent to which a successful communication of a target timbre in particular audio-visual conditions is reliant on the performer/piece. We found significant interaction results for the communication of *heavy* timbre (Figure 6), *sharp* timbre (Figure 7), and *velvety* timbre (Figure 8). The results of planned contrasts were labeled in these figures where significant three-way interaction was found. More specifically, P1 communicated *heavy* timbre better than P3 in aural conditions while P3 communicated better in the visual-only condition; P1 communicated *sharp* timbre better in the visual-only condition while P3 communicated better in aural conditions. In addition, P1 communicated *velvety* timbre more effectively in aural conditions than P2 whereas P2 was better in the visual-only condition.

To sum up, H3 was partially supported: the above results suggest that only the communication of *relaxed* and *tense* timbres is not reliant on the performer's differences. In other cases, there is an individual difference in the utilization of the audio-visual condition to communicate the timbral intention to the listeners. Each performer may be specialized in communicating a particular timbre with particular aural or visual cues.

### Non-verbal Sensory Judgment Analysis

Judgment of the size, shape, and brightness was collected as categorical data in the form of either: A (bigger size, rounder shape, and brighter version), or B (smaller size, sharper shape, and darker version) in the questionnaire. This data was replaced with a score of either 0 or 1, to give a method of calculating the mean across different participants (*N* = 21). The measure of each type of judgment (e.g., size evaluation while hearing *bright* timbre) for each participant was calculated *via* an average across AV stimuli and three music pieces (Mean = SUM divided by 9). Table 5 displays the value of the size, shape, and brightness evaluations in response to five pairs of timbre intentions. A paired sample *T*-test was conducted to compare the mean difference in non-verbal judgments in response to contrasting performances—e.g., the mean difference in size evaluation between hearing *bright* timbre and hearing *dark* timbre. Significant differences are shown with ^*^.

**Table 5:** Mean judgment scores for size, shape, and brightness in the perception of 10 timbres.

	**Size**	**Shape**	**Brightness**
	**(small) 0 – 1 (big)**	**(sharp) 0 – 1 (round)**	**(dark) 0 – 1 (bright)**
Bright	0.47^*^	0.45^**^	0.53
Dark	0.35^*^	0.62^**^	0.57
Round	0.49	0.66^**^	0.54
Sharp	0.59	0.17^**^	0.44
Heavy	0.58^**^	0.41^**^	0.53
Light	0.31^**^	0.72^**^	0.60
Tense	0.41	0.40^**^	0.48^*^
Relaxed	0.38	0.68^**^	0.63^*^
Dry	0.39	0.38^**^	0.54
Velvety	0.46	0.76^**^	0.57

** *p < 0.01*;

* *p < 0.05*.

The results indicate a strong timbre-shape association in all five pairs of timbral intentions, hence H4 was supported. Participants tended to choose the round object when hearing/seeing performances with *dark, round, light, relaxed*, and *velvety* timbral intentions and chose the sharp object when hearing/seeing performances with *bright, sharp, heavy, tense*, and *dry* timbral intentions. In contrast, size was associated with the perception of two pairs of timbres (*bright****/****dark* timbre, *heavy****/****light* timbre), and brightness was associated with only one timbre perception pair (*relaxed****/****tense* timbre).

### Principal Components Analysis (PCA) and Acoustic/Visual Information

The PCA revealed the presence of three components with eigenvalues >1, which account for a total of 69.6% of the variance in evaluations of the 10 timbres, explaining 38.71, 19.2, and 11.68% of the variance respectively. To aid the interpretation of the three components, varimax rotation was used and the results are displayed in Table 6. Loading values that were <0.3 were excluded from the table, so blanks in the table indicate where low loading exists.

**Table 6:** Factor loading after varimax rotation.

**Evaluation**	**Component 1**	**Component 2**	**Component 3**
	**Round**	**Heavy**	**Bright**
	**(Touch-movement)**	**(Weight-Negative intensity)**	**(Luminance)**
Round	**0.851**		
Velvety	**0.827**		
Dry	−0.626	0.333	0.340
Relaxed	0.595		0.486
Heavy		**0.811**	−0.328
Dark		**0.765**	−0.320
Tense	−0.473	0.650	
Sharp	−0.595	0.614	
Bright			**0.829**
Light		−0.344	**0.744**

*Bold values highlight the most two representative timbres in each dimension*.

*The highlighted red mark indicates the three clusters in PCA. They were framed for readability and highlighting*.

As a result, the first dimension (named as Round-dimension, eigenvalue: 38.7%) includes the timbre evaluation of round, velvety, relaxed (positive scores) and non-dry (negative score). This dimension is related to touch and movement qualities and is most strongly associated with Round. It is a combination of tactile feelings of roundness and velvetiness, and low dryness. The second component (named as Heavy-dimension) relates to the evaluation of heavy, dark, tense, and sharp. This is a combination of high intensity and negative valence and is associated with haptic sensations of sharpness and tactile sensations of heaviness. It is most strongly associated with Heavy and relates to experiences of weight and negative intensity. The last component (named as Luminance-dimension) is most strongly associated with Brightness followed by lightness. It seems that the Luminance-dimension is both positive in valence, high in space and lightweight.

The Appendix 4 displays the audio waves and video snapshot^5^ of the three pairs of performances (round vs. velvety, heavy vs. dark, bright vs. light) by three performers. We compared the aural/visual information in pairs, to explore whether the pianists were defaulting to the same performance style/gestures or not when playing these highly correlated timbres. Observable differences were found in these pairs:

Round vs. Velvety: P1 differentiated these two timbres using intensity (*round* is louder than *velvety*); while P2 used performance tempo to make contrast (*round* is quicker than *velvety*). In the visual cues, P1 and P2 changed sound-producing gestures, with higher hand position and curved finger to produce *round* timbre while using lower hand position and flatter finger to play *velvety* timbre. The difference in P3 was little.

Heavy vs. Dark: For P1 and P3, *heavy* timbre was interpreted as much louder than *dark* timbre. P2 played *slower* in dark timbre compared to *heavy* timbre. In general, all the three pianists were shown more solemn facial expression, and P3 showed more intensive feelings with inward upper-body movements toward the piano.

Bright vs. Light: In general, these two timbres were played much softer for the three performers. Differences were found in the facial expression and posture for P1 and P3, that *bright* timbre was played with more delighted and cheerful expression and movements.

## Discussion

### The Reliability of Communicating Timbre

The first research question examined in this study is whether the communication of timbral intention between pianists and listeners is reliable. The study results indicate that all the 10 timbres are communicated with an accuracy above chance level, although the average percentage correct was considerably higher for some timbres (*sharp, relaxed, and light*) than for others. Furthermore, the repeated-measures ANOVA results showed that nine of 10 timbres were reliably communicated, except for the *dark* timbre. This suggests that timbral intention can be communicated between the performer and the listeners like other abstract intentions including expressivity (Davidson, 1993), emotions (Juslin, 2000), and sensorimotor feelings (De Poli et al., 2017). There is already evidence demonstrating that instrument-expertise influences the perception of musical expressivity due to the activation of sensory representation in the observation of motor plan (Broughton and Davidson, 2014).

The difficulty of communicating the 10 timbres varies one from the other. *Bright* timbre was the easiest to communicate and not influenced by the AV condition factor or performer/piece, while *dark* timbre was the most difficult one and the communication even failed in the visual-alone condition. One of the potential reasons could be that this experiment uses a Yamaha piano which usually has a brighter sound, resulting in the superior communication of brightness.

**Figure 9:**
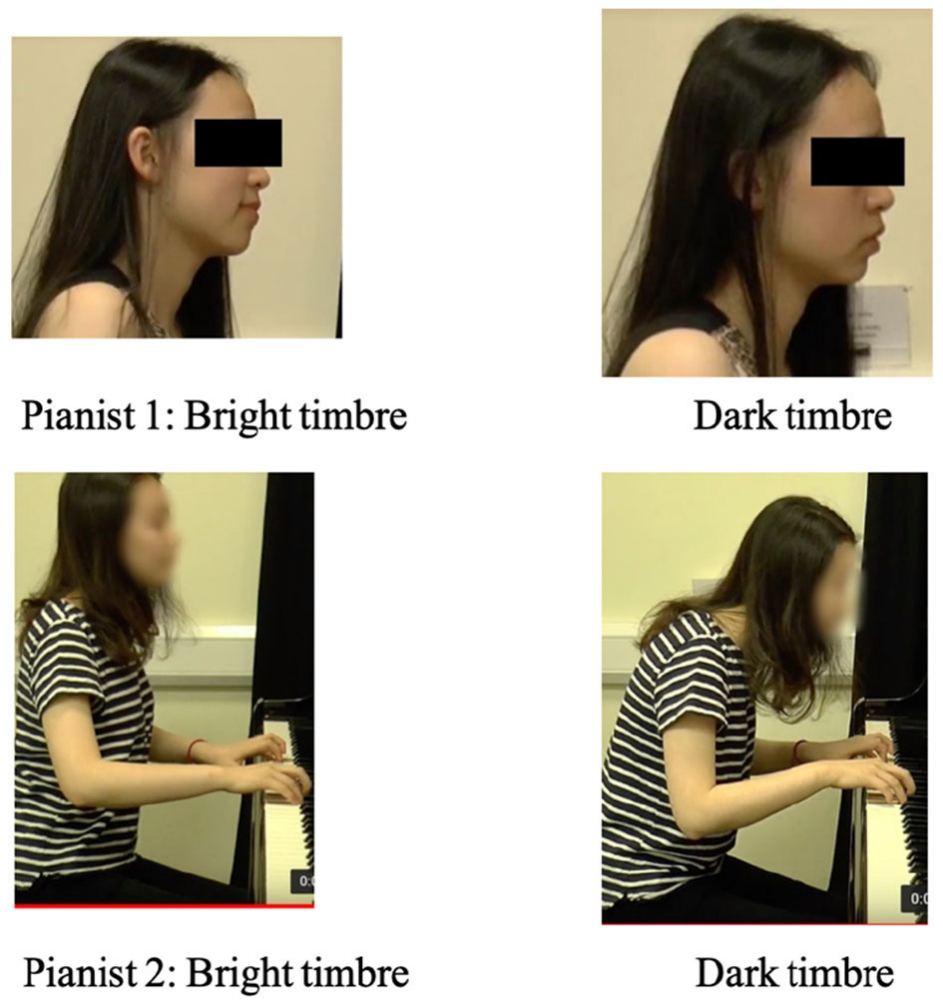
The facial expression and posture of two performers when expressing bright and dark timbres.

The differences in the communication outcome imply that music performers may consider suitable strategies (i.e., acoustic cues or visual cues) for the expression and communication of different timbres and that music educators are encouraged to focus on the teaching and learning of more difficult ones (i.e., *dark, round, and bright*) in piano lessons. Facial expression can be considered to differentiate *bright* and *dark* timbre, which has been demonstrated to be an effective way of differentiating the effects conveyed in the vocal performance (Thompson et al., 2005). For example, Figure 9 below shows that two performers in this research used different visual communication strategies to differentiate *bright* and *dark* timbre: one used facial expression while the other one used forward/inward upper-body movements.

### Hearing and Seeing Piano Timbre

The subsequent research question we investigated concerns the relevance of auditory and/or visual cues in the communication of timbral intention. Firstly, the comparison of sound waves across five pairs of timbres indicated that the inconsistency in the choice of other performance parameters (either using intensity or performance tempo) among the three pianists; however, the reliability of communicative process implied that the participants had a common, consensual understanding of the meaningful use of performance parameters to achieve certain timbre. This result is in line with Bernays and Traube's (2014) study that showed an individuality in the playing style of five pianists in the expression of the same timbral intention, with a personalized choice of musical features (articulation, pedal, attack, dynamics, etc.).

Secondly, the importance of visual cues in music performance is verified for the paring of *relaxed* and *tense* timbres, where visual information determined the effectiveness of timbral communication independent of the pianist/piece. For the remaining timbres (*heavy, light, round, sharp, dry, and velvety*), the influence of the AV condition was in most cases accompanied by an interaction involving the effect of pianist/piece (e.g., when communicating *sharp* timbre, P1 is better in visions while P3 is better in sounds), implying the difference in the preference of using visual cues among the pianists. Interestingly, increasing numbers of scholars (Thompson et al., 2005; Schutz and Lipscomb, 2007; Schutz, 2008; Davidson, 2012) have started to claim the benefits of using bodily communication in a music performance for the performers, including enhancing auditory experience, shaping the perception of musical notes, or facilitating the comprehension of lyrics. According to Schutz (2008), percussive instruments such as the piano and marimba could be most successful in benefiting from visual communication due to limited timbral nuances and percussive/short note length. We therefore encourage pianists to use bodily communication (i.e., appropriate facial expressions, gestures, and postures) in the communication of timbral intention, in addition to the auditory component of the music performance.

We integrated two angles including sound-producing gestures and ancillary gestures in the video stimuli. Although it is unknown whether the visual communication of 10 timbres differs in the reliance on specific aspects of bodily movements, we anticipate that the visual information of sound-producing gestures underlies the perception of 10 timbres—seeing how the keyboard is touched (i.e., percussive/non-percussive, curved/flat, hard/soft, etc.) influenced the listeners' perception of piano timbre, as suggested by the McGurk effect in music perception (Saldaña and Rosenblum, 1993; Schutz and Lipscomb, 2007). Further study needs to be taken to investigate the impact of ancillary gestures on timbre perception.

Last but not the least, the successful communication of timbral intention even in the vision-alone condition suggests an embodied perspective in music communication. One possible explanation is that understanding a pianist's silent movements possibly involves the simulation of a performative action in the listener's head, which leads to the triggering of an auditory image of the timbral effect (Keller, 2012). This is in line with previous studies (Camurri et al., 2003; Dahl and Friberg, 2007) showing that listeners can still detect the emotional intention of performers even when the sound information is unavailable. Brain imaging studies supported this and found that there are activations of certain motor-related areas in the brain when imagining music with the “inner ear” (Zatorre and Halpern, 2005) and in the perception of musical sounds (Haueisen and Knösche, 2001). This study extends the view of embodied music listening, by showing the successful communication of timbral intentions even without aural cues.

In this study, the listeners can “stand in someone else's shoes” to understand the actions, intentions, and sounds of the pianists by simulating the motor activity. According to Molnar-Szakacs and Overy (2006), a “similar or equivalent motor network is engaged by someone listening to singing/drumming as the motor network engaged by the actual singer/drummer” (p. 236) and therefore the communication of timbre was also successful in audio-alone conditions due to a simulation of a motor activity similar or equivalent to that associated with the timbral effect. The loop between sensory experience and motor commands has been explained by the “internal model” in the research into sensorimotor perceptions (Keller, 2012; Maes et al., 2014), which suggests its benefits for musicians themselves including action planning and self-monitoring (Novembre and Keller, 2014) and the benefits for co-players such as synchronization and coordination (Keller et al., 2007). This study demonstrated the benefits of an internal model in performer-listener communication, which is a shared understanding of actions and sounds that occur in the pianists' communicative process of timbral intentions to the listeners. We, therefore, suggest that the listeners are also performers because a mirror process happens in the perception of piano timbre through the encoding of expressive gestures into sounds (timbres) and the decoding of sounds (timbres) into expressive gestures (cf. Leman and Maes, 2014).

### Timbre-Related Cross-Modal Correspondence

The results of non-verbal sensory judgement indicated that a cross-modal timbre-shape association was found in all 10 timbres. When music was played with *dark, round, light, relaxed*, and *velvety* timbral intentions, it was felt to be rounder than when played with *bright, sharp, heavy, tense*, and *dry* intentions. This timbre-shape association is in line with several previous studies which have found a sharpness/color association with either *soft* or *harsh* timbres (Adeli et al., 2014) as well as visual textural associations (Giannakis, 2006). In addition to a timbre-shape association, this study also found a timbre-size association in two pairs of timbres (*bright****/****dark, heavy****/****light*).

Wallmark and Kendall (2018) applied the theory of conceptual metaphor (Lakoff and Johnson, 1980) to understand timbre semantics. They explained that when timbre is described with light contrast (e.g., bright/dark) and textural feature (e.g., rough, smooth), it reflects the conceptual metaphors that SOUND IS LIGHT and SOUND IS TEXTURE as people use the source domain of vision and tactile sensations as a reference to drive the meaning of the target domain (i.e., timbre). This statement reflects how abstract domains of human experience can be understood by concrete, embodied accounts. The embodied accounts to explain timbre-related CMC can also be found in Spence (2011) who provided three for the underlying reasoning of CMC: statistical, semantic-mediated, and embodied accounts. Statistical and embodied accounts explained the results of repeated exposure to, and physical interaction with, instruments and the extent to which the weak synaesthesia experience related to piano timbre can be established as a part of musical training (Spence, 2011). Studies have indicated that repeated exposure to statistical co-current pairs of stimuli can help to shape the coupling pairs, even in circumstances where the stimuli are unrelated [e.g., the association between a feeling of stiffness and visual luminance, Ernst (2007)]. Semantic-mediated accounts help to understand the linguistic descriptions related to piano timbre and their possible impact in generating a cross-modal coupling experience [cf. Dolscheid et al. (2013)]. For example, in the production of round timbre, a teacher's verbal description (round), modeling, and the embodiments (round handshape) may help the student to establish an association between the sound outcome and the round shape.

### Piano Timbre as a Component of Expressivity

For a long time, the definition of musical expression/expressiveness has been deeply influenced by Seashore's 1938 statement on “deviation from the score”^6^ [see a discussion in Clarke and Doffman (2014)]. The shortcomings of this definition and the over-emphasis on the sonic properties of performance have been noted by several scholars, for instance leading to a trend of the musical score being the primary ontological focus of music (Dogantan-Dack, 2014) and a disembodied, ahistorical account of musical performance by conceptualizing the score as the piece (Clarke, 2004). This research would like to assure that, in the pursue of timbral nuances, musical expressiveness is imprinted in the acoustic variance and the subtle control of gestures and touch of pianists, as if “touch is the expressive skeleton on which the pianist enfolds the expressive flesh” (Dogantan-Dack, 2014, p. 7). Touch, gestures, bodily movements in piano playing actively shape a listener's multimodal perception of piano timbre, resulting in sensorimotor perception and cross-modal correspondences.

## Implications and Conclusions

There are some implications for instrumental music pedagogy and performance. This study offers interesting insights into the question of “what do listeners perceive in timbre in music performance.” From the listener's perspective, the perception of timbre is not merely variations in acoustic information but related to multimodal perception including kinaesthetic and muscular sensations (heavy-light, tensed-relax) and cross-modal correspondences (physical size and shape). This is consistent with the studies of the semantics of timbre (Bellemare and Traube, 2005; Bernays, 2013; Kojucharov and Rodà, 2015; Saitis and Weinzierl, 2019) that have shown rich subjective experiences and multidimensionality in response to timbre. Therefore, music teachers and students are encouraged to use cross-domain metaphors and multimodal communications (gestures, modeling, touch, etc.) in the teaching and learning of timbre-related performance goals in music lessons (Li and Timmers, 2021).

This study suggests that timbral intention can work well as a bridge that connects the performer and the listener, due to the resonance of sensorimotor knowledge induced by musical sounds. It also has implications for music education that teachers should encourage students to mobilize timbre as a deeper motivation for expressing intention/message and communicating it to their listeners. As Dogantan-Dack (2017) pointed out, classical musicians are facing pressure due to the sustainability of the profession itself and the diversity of today's musical genres and practices. In the process of working on timbral intentions and communicating them to the listeners, student-pianists may increase their musical competency and sense of autonomy [i.e., expressive freedom extending beyond the composer's intention, Dogantan-Dack (2017)], which may help to fulfill their psychological needs at this stage of their musical education and maintain the motivation to persist and engage with music (Wise et al., 2017).

One of the limitations in this study is the unsystematic control of performance parameters such as dynamics and tempo, and we acknowledge that these elements can influence musical perception. This enables a more open musical interpretation for the performers to express different timbral intentions, which probably work as co-variables to influence the listeners' judgments. Another limitation is the limited sample size used in the present study; thus, the results of the variety in communication outcomes of 10 timbres must be taken as tentative. Future research on the visual communication of piano timbre may consider using less timbres, for instance relaxed/tense and heavy/light, to expand our understanding of touch qualities (e.g., soft/hard, percussive/non-percussive) with deeper insights into bodily feelings in piano playing. Alternatively, future research may also consider using non-pianists as the control group (i.e., the level of musical training as the between-group factor). There is already evidence demonstrating that instrument-expertise influences the perception of musical expressivity due to the activation of sensory representation in the observation of motor plan (Broughton and Davidson, 2014). Future research can consider more restricted control of performance parameters using a single piano tone or chord. Furthermore, future research may be able to use point-light displays to replace bodily movement in the videos, to avoid the influence of familiarity of performers or their facial expressions. This method could also help to create congruent or incongruent stimuli, by synchronizing the movement features of one stimulus with the performance data of another in terms of onset/offset time, duration, dynamics, and pedaling [cf. Vuoskoski et al. (2014)]. For example, a new artificial video can be generated *via* synchronizing movement features in a “tense” performance with the sound signal of a “relaxed” performance thereby allowing an examination of the extent to which visual information influences or modifies listeners' judgments.

## Data Availability Statement

The raw data supporting the conclusions of this article will be made available by the authors, without undue reservation.

## Ethics Statement

The studies involving human participants were reviewed and approved by the University of Sheffield. The participants provided their written informed consent to participate in this study. Written informed consent was obtained from the participants for the publication of any identifiable images or data included in this article.

## Author Contributions

SL and RT designed the experiment. SL was responsible for data collection, data analysis, and the draft of manuscript. RT and WW contributed to refine arguments, the presentation of results, and to improve the readability. All authors contributed to the article and approved the submitted version.

## Funding

This research was funded by Chinese Postdoctoral International Exchange Program [grant number 273060] and Research on adolescent internet adaptation-oriented optimization method for personalized information service [NSFC Grant No. 71974072].

## Conflict of Interest

The authors declare that the research was conducted in the absence of any commercial or financial relationships that could be construed as a potential conflict of interest.

## Publisher's Note

All claims expressed in this article are solely those of the authors and do not necessarily represent those of their affiliated organizations, or those of the publisher, the editors and the reviewers. Any product that may be evaluated in this article, or claim that may be made by its manufacturer, is not guaranteed or endorsed by the publisher.

## Footnotes

^1^See Li and Timmers (2021) and Li et al. (2020) for a detailed explanation of the similarities and differences in these terminologies.

^2^The validity of using the five timbre descriptors as performance instructions has been tested in their study with sound analysis and measurements of performance strategies (e.g. attack depth, speed, decay, etc.) by five pianists. Their study indicated that there was no difficulty in pianists interpreting the same musical piece with distinct timbral intentions, and subtle changes in expressing different timbres were found by sound and keyboard motion analysis.

^3^The method of calculating absolute and relative scores of percent correct answers is borrowed from Timmers and Ashley (2007, Experiment 2).

^4^Timbre metaphors will now be italicized for better readability and display of the results.

^5^Video snapshots were captured at the same time slot to obtain a relatively fair comparison. However, video shortcuts failed to provide dynamic and continuous gestural changes e.g. how fingers are articulated (smooth or detached).

^6^which defines expression as a deviation from the notated values (i.e. pure tone, true pitch, even dynamics, rigid rhythm etc.) on the musical score.

## Supplementary Material

Supplementary Video 1 P1_bright.

Supplementary Video 2 P1_dark.

Supplementary Video 3 P1_heavy.

Supplementary Video 4 P1_light.

Supplementary Video 5 P1_relax.

Supplementary Video 6 P1_tense.

Supplementary Video 7 P1_round.

Supplementary Video 8 P1_sharp.

Supplementary Video 9 P1_dry.

Supplementary Video 10 P1_velvety.
