# Supplementary material for: The Communication of Timbral Intentions Between Pianists and Listeners and Its Dependence on Auditory-Visual Conditions
Source: Front Psychol. 2021 Sep 21;12:717842. doi: 10.3389/fpsyg.2021.717842 (PMC8491637; doi:10.3389/fpsyg.2021.717842)
Supplement: Supplementary file 1 [file Data_Sheet_1.pdf]

Appendix 1

Music score used in the perceptual experiment

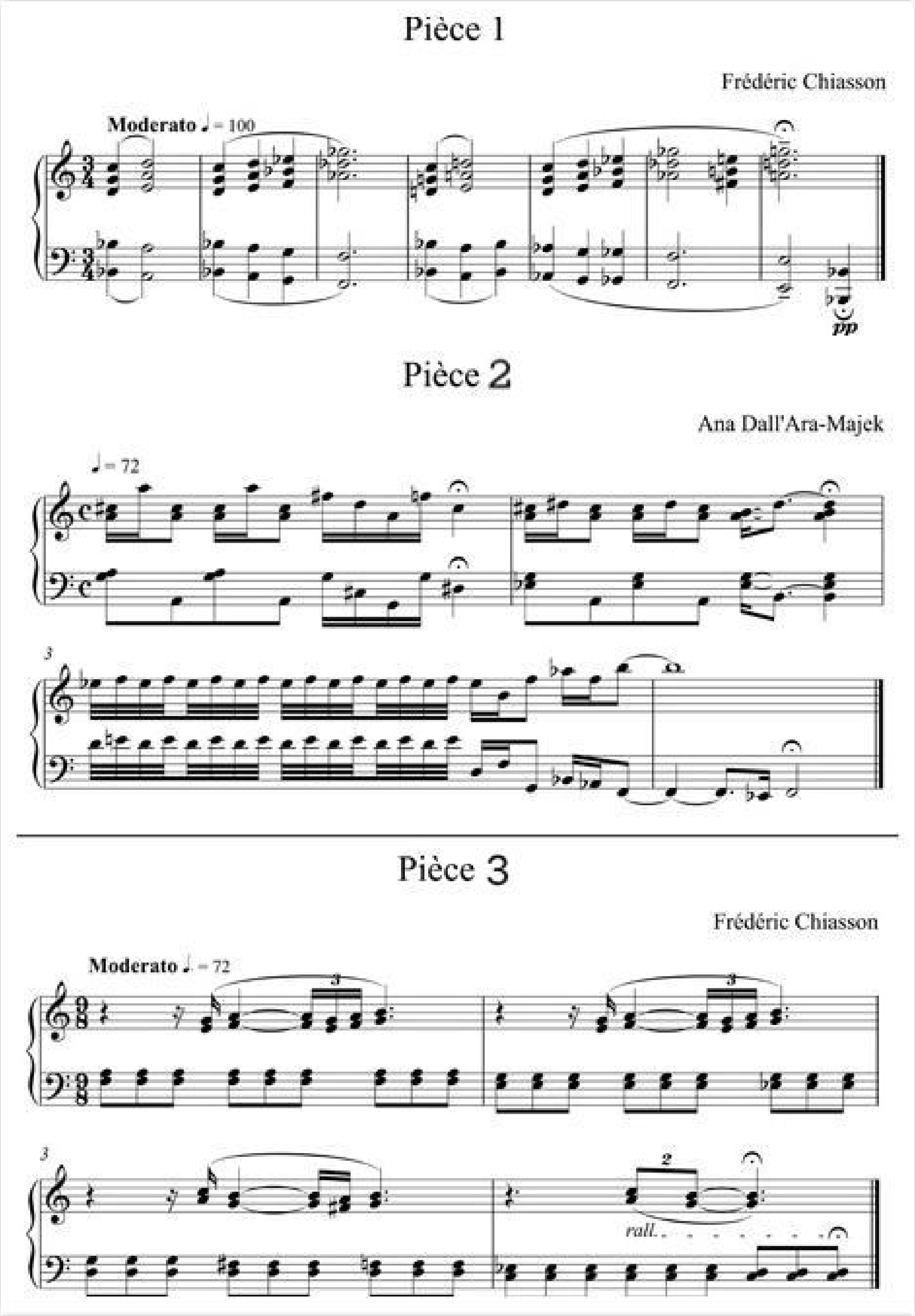

Appendix 2

Response Sheet for Listeners in the perceptual experiment

Excerpt No. (   )__

请根据您所听到的音乐/视频片段, 回答下列问题 *Please listen to the music extract, and answer the following questions*

1. 我认为钢琴家想表达的音色为：*(I think the timbre expressed by the performer is)*

	非常不同意 *Strongly disagree*			适中 *Moderate*			非常同意 *Strongly agree*
紧张的 Tensed	1	2	3	4	5	6	7
放松的 Relaxed	1	2	3	4	5	6	7
轻盈的 Light	1	2	3	4	5	6	7
沉重的 Heavy	1	2	3	4	5	6	7
明亮的 Bright	1	2	3	4	5	6	7
阴暗的 Dark	1	2	3	4	5	6	7
圆润的 Round	1	2	3	4	5	6	7
尖锐的 Sharp	1	2	3	4	5	6	7
干燥的 Dry	1	2	3	4	5	6	7
天鹅绒般的 Velvety	1	2	3	4	5	6	7

2. 我认为钢琴家所诠释的音色，符合下列图像中的这一个：（请在每组中选取一个）*I think the timbre expressed by the performer is in accordance with the figure of: (please choose either A or B in each pair)*

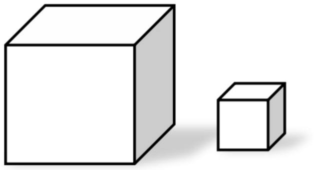

A.　　B.

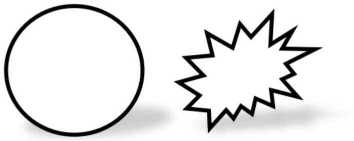

A.　　B.

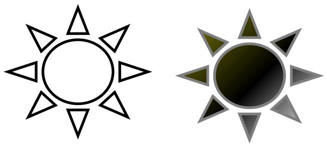

A.　　B.

Appendix 3

Information Sheet for the Performers in Study 2 (perceptual experiment)

*You are being invited to help the researcher to create the experimental stimuli for her listening experiment on '**the perception of piano timbre**'. Please take time to read the following information and decide whether or not you wish to participate. Please also ask the researcher if you would like more details about the research. Thank you for taking the time to read this information.*

The Project

This research aims to explore the role of visual information of musical performance in the perception of piano timbre among listeners, which forms a part of researcher's PhD research project.

Why have I been selected to be involved in this project?

You are being invited to take part due to your expertise in piano performance or teaching experience which meets the requirement of participation in this research (ABRSM grade 8 or equalized).

What are the performance tasks about?

You will be asked to play three musical pieces in **ten timbres (tense/relaxed, heavy/light-weight, bright/dark, round/sharp, and dry/velvety)**. You will be both video-recorded with two HD camcorders capturing your entire body and finger movements separately as well as audio-recorded using the MIDI piano.

The participants in the listening experiment will be presented with your performance in three different ways (heard-only, seen-only, or both heard and seen) and asked to **rate their judgement of perceived timbral qualities**.

More information or questions?

If you would like more information about, or have a concern or question about the project, please contact Shen Li (sli37@sheffield.ac.uk).

Appendix 4
Sound Waves and Visual information

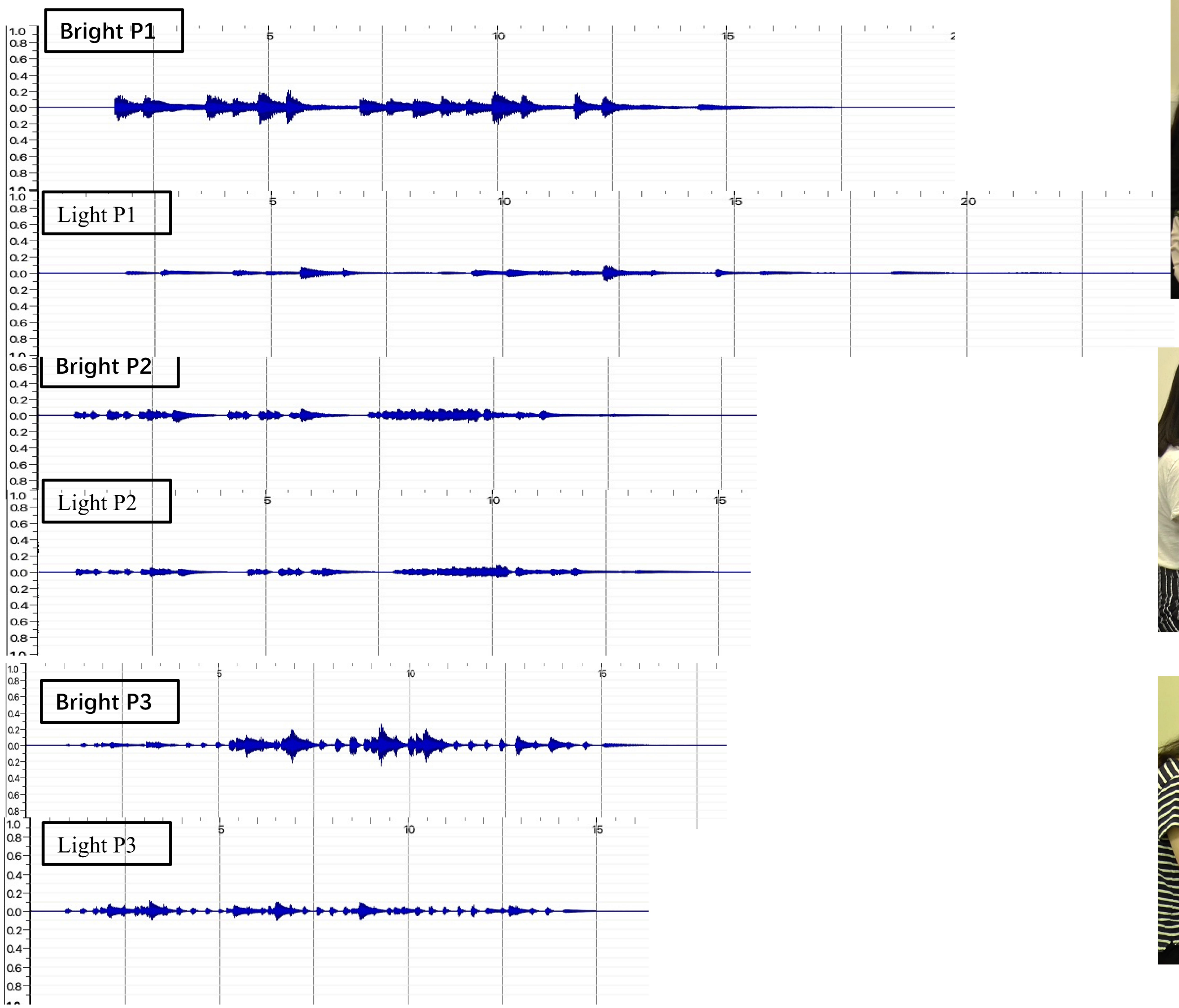

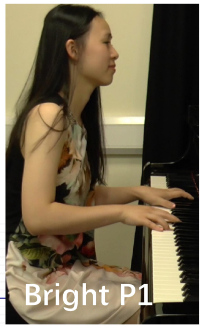

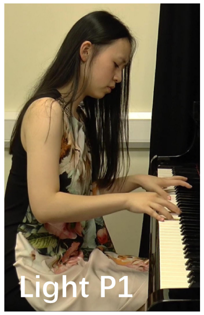

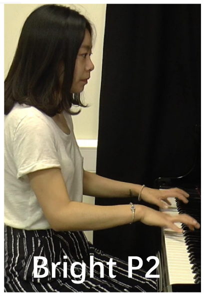

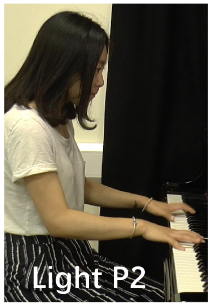

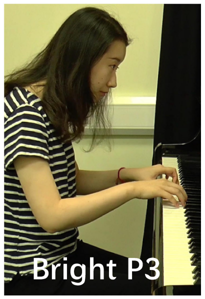

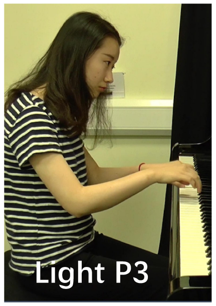

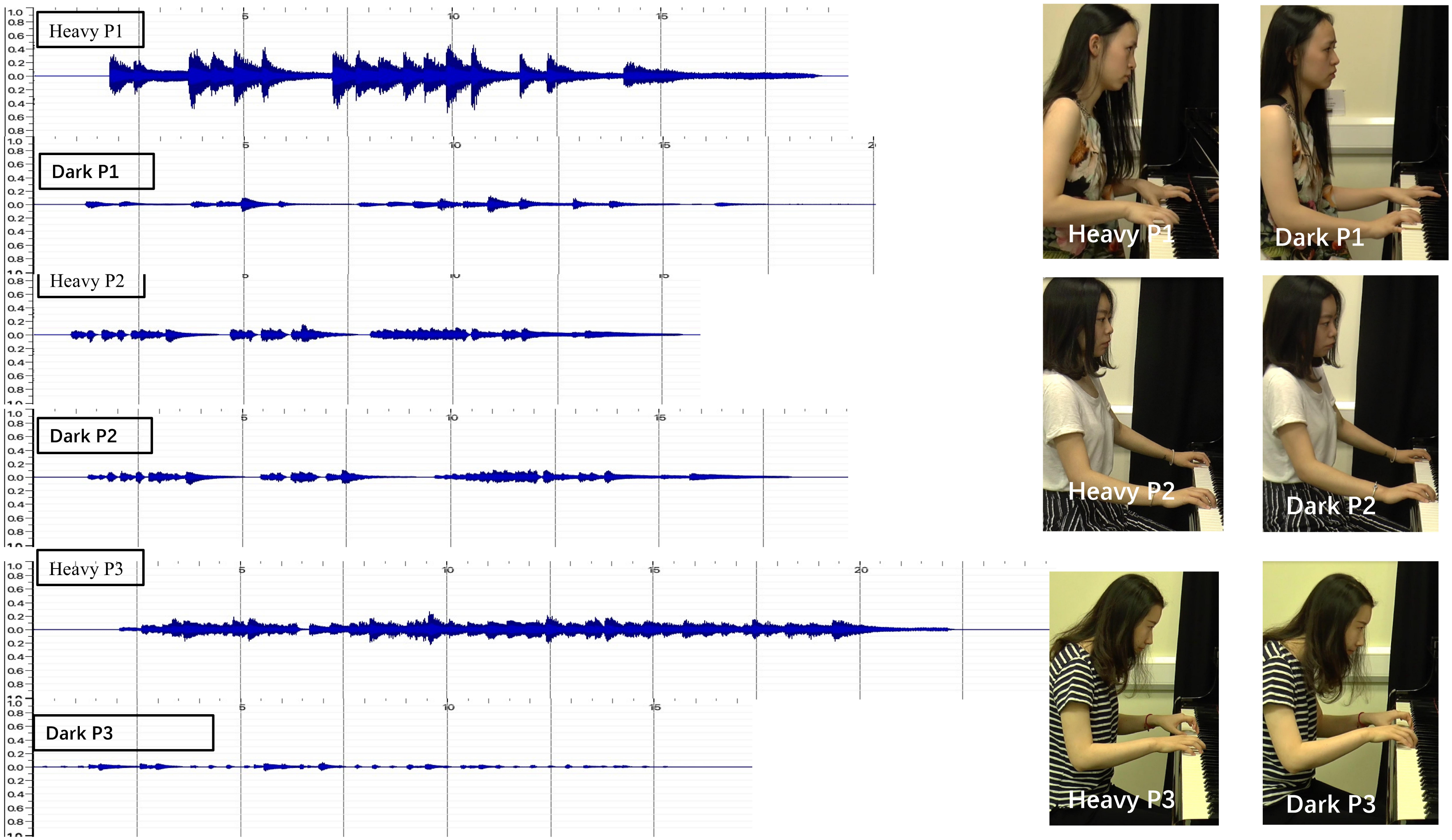
Heavy P1
Dark P1
Heavy P2
Dark P2
Heavy P3
Dark P3
Heavy P1
Dark P1
Heavy P2
Dark P2
Heavy P3
Dark P3

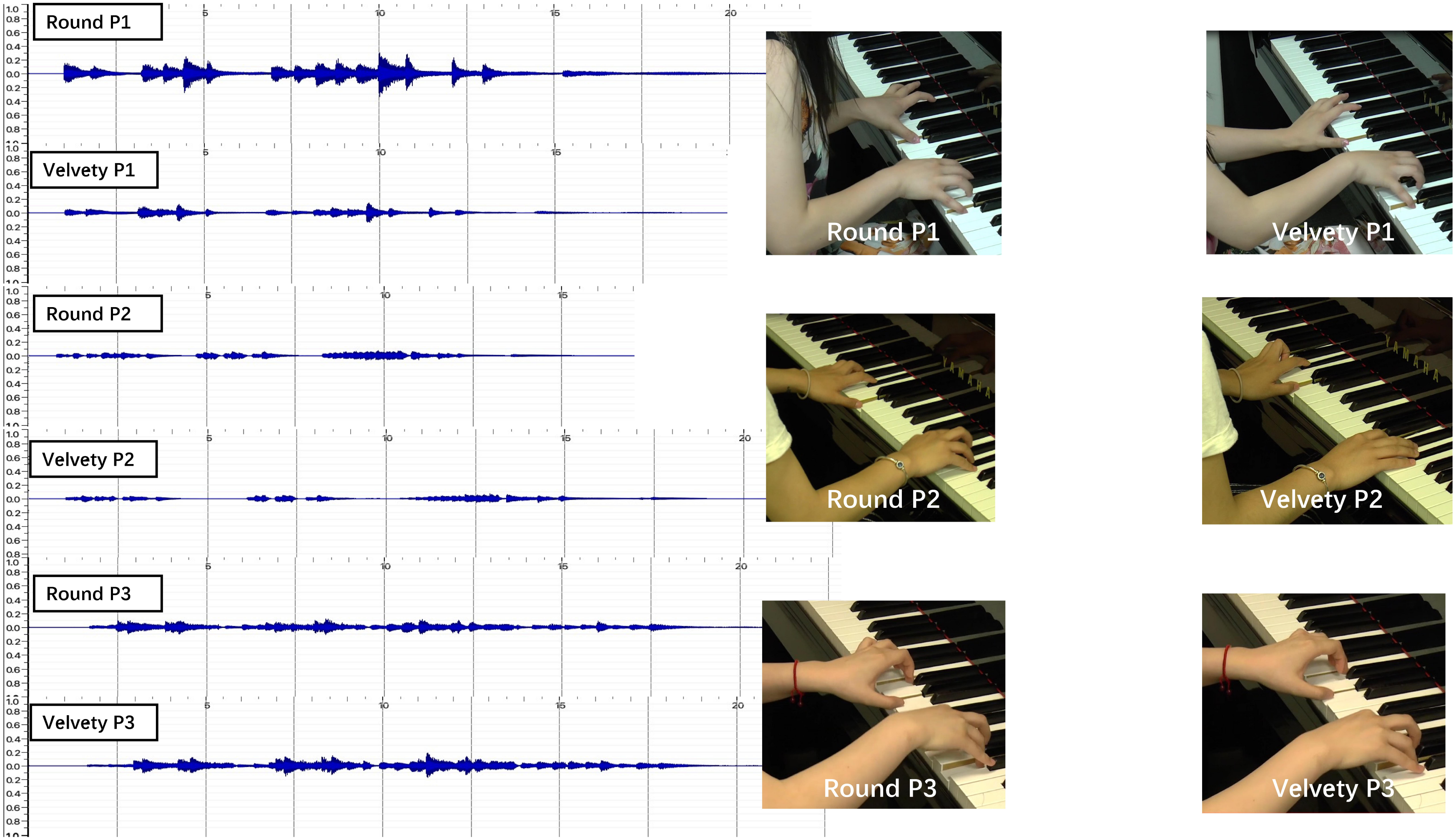
Round P1
Velvety P1
Round P2
Velvety P2
Round P3
Velvety P3
Round P1
Velvety P1
Round P2
Velvety P2
Round P3
Velvety P3
